# Risk Factors of Correction Loss After Percutaneous Reduction and Fixation for Thoracolumbar Burst Fracture: A One-Year Follow-Up Study

**DOI:** 10.7759/cureus.84961

**Published:** 2025-05-28

**Authors:** Masato Tanaka, Aditya Thakur, Muhamad A Rahman, Akshay Fuse, Shinya Arataki, Tadashi Komatsubara, Akiyoshi Miyamoto, Masakazu Nagamatsu, Tomoyoshi Sakaguchi

**Affiliations:** 1 Department of Orthopaedic Surgery, Okayama Rosai Hospital, Okayama, JPN; 2 Department of Radiology, Okayama Rosai Hospital, Okayama, JPN; 3 Department of Rehabilitation, Okayama Rosai Hospital, Okayama, JPN

**Keywords:** correction loss, focal kyphtic angle, hounsfield unit, long fixation, osteoporosis, percutaneous reduction and fusion, short fixation, thoracolumbar burst fracture, vertebral comminution, vertebral wedge angle

## Abstract

Study design and purpose: This is a single-center retrospective observational study. The study aimed to find out the risk factors for correction loss after percutaneous reduction and fixation for thoracolumbar burst fractures.

Materials and methods: This study included 25 patients who underwent percutaneous reduction and pedicle fixation for thoracolumbar burst fractures from 2017 to 2024. Radiographic assessments were performed to identify vertebral wedge and focal kyphosis angles pre-operatively, post-operatively, and at one-year follow-up for all patients. Then, patients were divided into two groups: no correction loss (Group NCL), which had <5 degrees of correction loss, and correction loss (Group CL), which had 5 and >5 degrees of correction loss at one-year follow-up. Between the two groups, radiological parameters, BMI, osteoporosis, long/short construct, surgical time, intraoperative blood loss, postoperative complications, and revision surgery rate were evaluated. In comparing the groups, the Mann-Whitney U test analysis was used for continuous variables, while the Fisher exact test was used for dichotomous variables.

Results: The pre-operative wedge angle was statistically greater in Group CL (23.1 ± 6.8, 17.8 ± 6.7 degrees, *p*<0.001, mean difference 5.3, 95% confidence interval 19.7, 26.5). The final vertebral wedge angle in Group NCL statistically improved and was maintained at the final follow-up (*p*<0.001). The post-operative vertebral wedge angle in Group CL was significantly improved post-operatively (*p*<0.001), but decreased at final follow-up (p<0.001). The BMI, osteoporosis, long/short construct, surgical time, intraoperative blood loss, and postoperative complications were not significantly different. No revision surgery was observed in either group.

Conclusions: Percutaneous reduction and fixation for thoracolumbar fractures could correct and maintain good spinal alignment. The preoperative large vertebral wedge angle was the only risk factor for correction loss. Short fixation, osteoporosis, and vertebral comminution were not significant risk factors in our study.

## Introduction

Thoracolumbar burst fractures are one of the most common types of spinal injury, defined as fractures that disrupt both the anterior and middle columns of the vertebrae, often resulting from high-energy trauma such as falls or motor vehicle accidents [1]. These injuries can be classified as stable or unstable based on the integrity of the posterior ligamentous complex and neurological deficits. Stable burst fractures typically allow for conservative management due to their preserved structural stability. Conservative treatment for stable burst fractures involves immobilization, pain relief, and gradual mobilization, often with the use of a thoracolumbosacral orthosis (TLSO). While this approach can be effective for many patients, failure remains a concern in certain cases due to risk factors such as advanced age, increased interpedicular distance, and kyphotic deformity [2]. These failures may lead to the need for surgical intervention to address progressive neurological deficits or deformities that develop during follow-up [3].

Unstable fractures may necessitate surgical intervention to prevent progressive deformity or neurological compromise [4]. If the surgical intervention is indicated, approaches to thoracolumbar burst fractures can be categorized into traditional open surgery reduction and fusion with or without decompression and minimally invasive surgery (MIS). Open surgery often offers direct access for comprehensive decompression and stabilization but is associated with higher perioperative morbidity and extended recovery times [5]. In contrast, MIS techniques, such as percutaneous pedicle screw fixation with a reduction system, provide comparable outcomes with the advantages of reduced access-related morbidity, smaller incisions, reduced blood loss, shorter hospital stays, and faster postoperative recovery [5].

One of the main concerns is correction loss after fracture reduction. Previous studies have reported that the risk factors for correction loss were patient-specific conditions such as osteoporosis [6], body mass index (BMI) [7], and fracture morphology factors such as large vertebral wedge angle [8], McCormack and Gaines load-sharing classification score [9], disc and endplate injury status [10]. Others were surgical factors like increased blood loss and surgical time [11], short fixation [12], and greater correction of focal kyphosis magnitude [13]. This study aims to evaluate the association of these factors with correction loss following percutaneous reduction and fixation for thoracolumbar burst fractures.

## Materials and methods

This study has been approved by the Ethics Committee of Okayama Rosai Hospital (No. 547). The necessary informed consents were duly signed and obtained from all the patients involved in the study. A single-center retrospective analysis of patients who underwent posterior percutaneous reduction and fixation for thoracolumbar burst fractures between 2017 and 2024. Inclusion criteria included (1) thoracolumbar single burst fractures from T11 to L2; (2) percutaneous reduction and fixation; (3) age greater than 18 years. Exclusion criteria included (1) less than one-year follow-up; (2) ankylosed spine, metastatic tumor, rheumatoid arthritis, infection; (3) lack of data. A total of 29 patients were operated on, and 25 matched the inclusion criteria.

**Figure 1:**
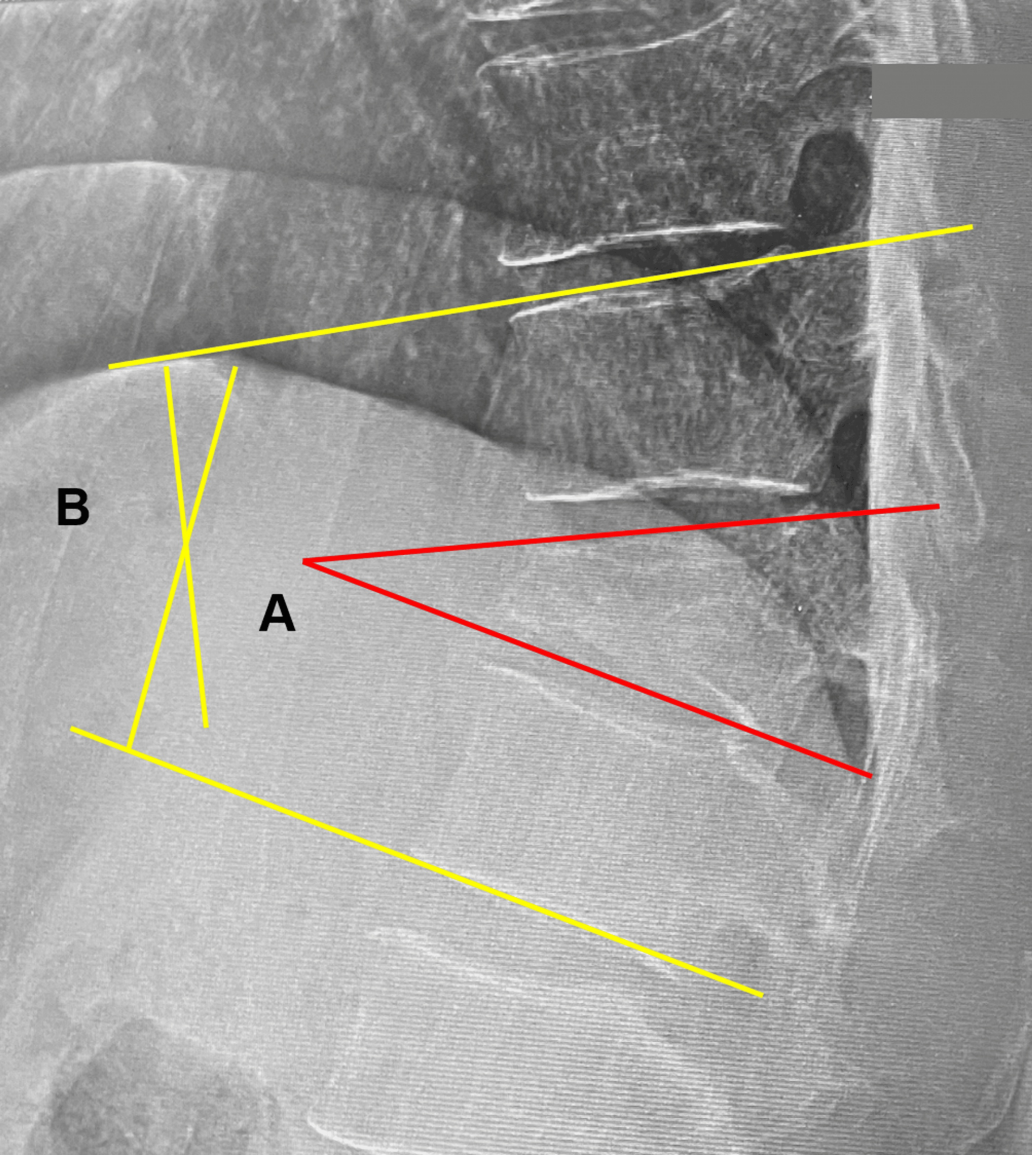
Radiological parameters. A: Vertebral wedge angle, B: Focal kyphotic angle. The radiograph is from our study group.

Radiographic assessments have been performed on all patients to identify vertebral wedge and focal kyphosis angles pre-operation, post-operation, and at one-year follow-up (Figure 1). AO (Arbeitsgemeinschaft für Osteosynthesefragen) classification [14], surgical construct, and implant failure were also evaluated.

The McCormack and Gaines load-sharing score and osteoporosis by Hounsfield units were assessed using a CT scan. MRI study evaluated the spinal cord, disc, and endplate injury status. The patient's neurological status was assessed using the International Standards for Neurological Classification of Spinal Cord Injury (ISNCSCI), American Spinal Injury Association (ASIA) score [15]. Patients were divided into two groups: Group NCL (No Correction Loss), which had <5 degrees of correction loss, and Group CL (Correction Loss), which had 5 and >5 degrees of correction loss at one-year follow-up. The surgical outcomes were measured by evaluating surgical technique, surgical time, intraoperative blood loss, postoperative complications, and revision surgery rate between the two groups.

Statistical analysis

The normality of continuous variables was assessed using the Shapiro-Wilk test, and the Student t-test or Mann-Whitney's U test was used to compare continuous variables between two groups. Categorical variables were analyzed using Chi-square or Fisher exact test to identify non-random associations. All statistical calculations were meticulously performed using GraphPad Prism version 6.0 (GraphPad Software, La Jolla, CA, USA). Statistical significance was set at p<0.05, with this threshold guiding the identification of meaningful differences and associations within the study's findings.

Surgical technique

**Figure 2:**
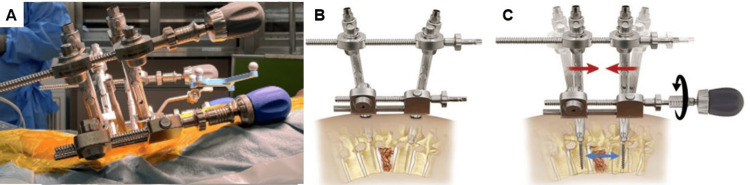
Surgical technique. A: Intraoperative image, B: Schematic diagram before reduction, C: Schematic diagram showing reduction. The permission of schematic diagrams of B and C have been taken from Medtronic.

All the surgeries were performed under general anesthesia with O-arm (Medtronic, Minneapolis, MN, USA) with navigation and without using C-arm fluoroscopy (Zenition 50, Philips, Amsterdam, Netherlands). Minimal invasive posterior stabilization with the trauma system Longitude®/Solera® system (Medtronic, Minneapolis, MN, USA) was done. Once the instruments were registered with the system, pedicle screws were inserted in the level above and below the fractured level. Rods are inserted and secured with set screws. Initially, some distraction is achieved with a lengthening device, then the angulation is corrected. With this trauma system, the lengthening device, which remains outside the skin, becomes the hinge point of reduction. When the angular reduction (red arrows) is performed with an angulation correction device, this mechanism prevents the bony fragment from protruding during reduction and acts as a distraction force of ligamentotaxis. After angular reduction is achieved, radiograms are taken to check alignment. If the reduction is insufficient, another distraction or angulation force can be applied carefully to avoid too much lengthening. Final tightening of set screws is done, extenders are removed (Figure 2).

Case illustration

**Figure 3:**
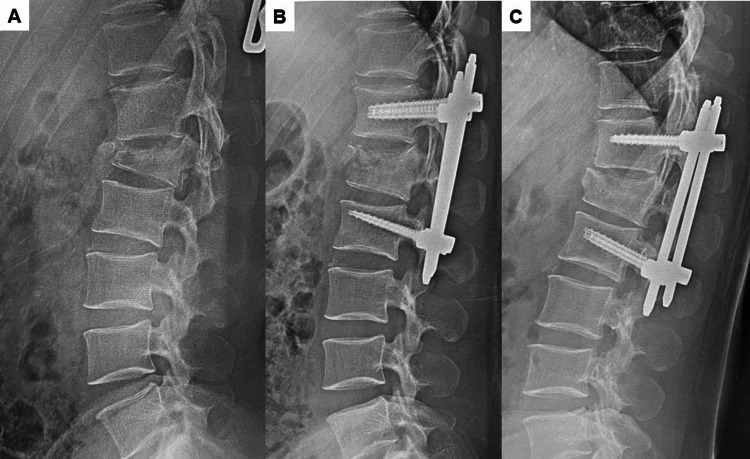
Case illustration from our study group; 33-years-old male individual, L1 burst fracture, American Spinal Injury Association (ASIA)-E, T12-L2 percutaneous reduction and fixation. A: Pre-operative lateral radiogram, B: Post-operative lateral radiogram, C: Final follow-up lateral radiogram.

The patient was a 33-year-old male individual with an L1 burst fracture. He had no neurological deficit and underwent T12-L2 percutaneous reduction and fixation with the trauma system (Figure 3). The post-operative fracture reduction was acceptable, but slight correction loss was observed at the final follow-up.

## Results

Patient demographics

The median ages in Group NCL and Group CL were 66 (22-83) and 36 (19-56) years, respectively. The age of Group NCL was younger than that of Group CL (*p*=0.042). There was no significant difference in gender between the two groups (*p*=0.131). BMI, fracture level, and neurological status were also not significantly different (Table 1).

**Table 1:** Patient demographics. Fisher exact test was used for gender, fracture level, and neurological status. Mann-Whitney U test was used for age, and body mass index. NCL: no correction loss, CL: correction loss.

	Group NCL	Group CL	Statistical test	Test value	p value
Gender (female/male)	7/8	2/8	Fisher exact	-	0.229
Age (years): Median	66 (22-83)	36 (19-56)	Mann-Whitney U	35	0.042
Body mass index (kg/m^2^)	21.0 ± 4.5	22.7 ± 4.2	Mann-Whitney U	47	0.62
Fracture level	T11:1, T12:4, L1:7, L2:3	T11:0, T12:2, L1:6, L2:2	Fisher exact	-	1
Neurological status	A:2, B:0, C:1, D2, E:10	A:0, B:0, C:3, D:0, E:7	Fisher exact	-	0.327

Comparison of the radiological parameters between the two groups

**Figure 4:**
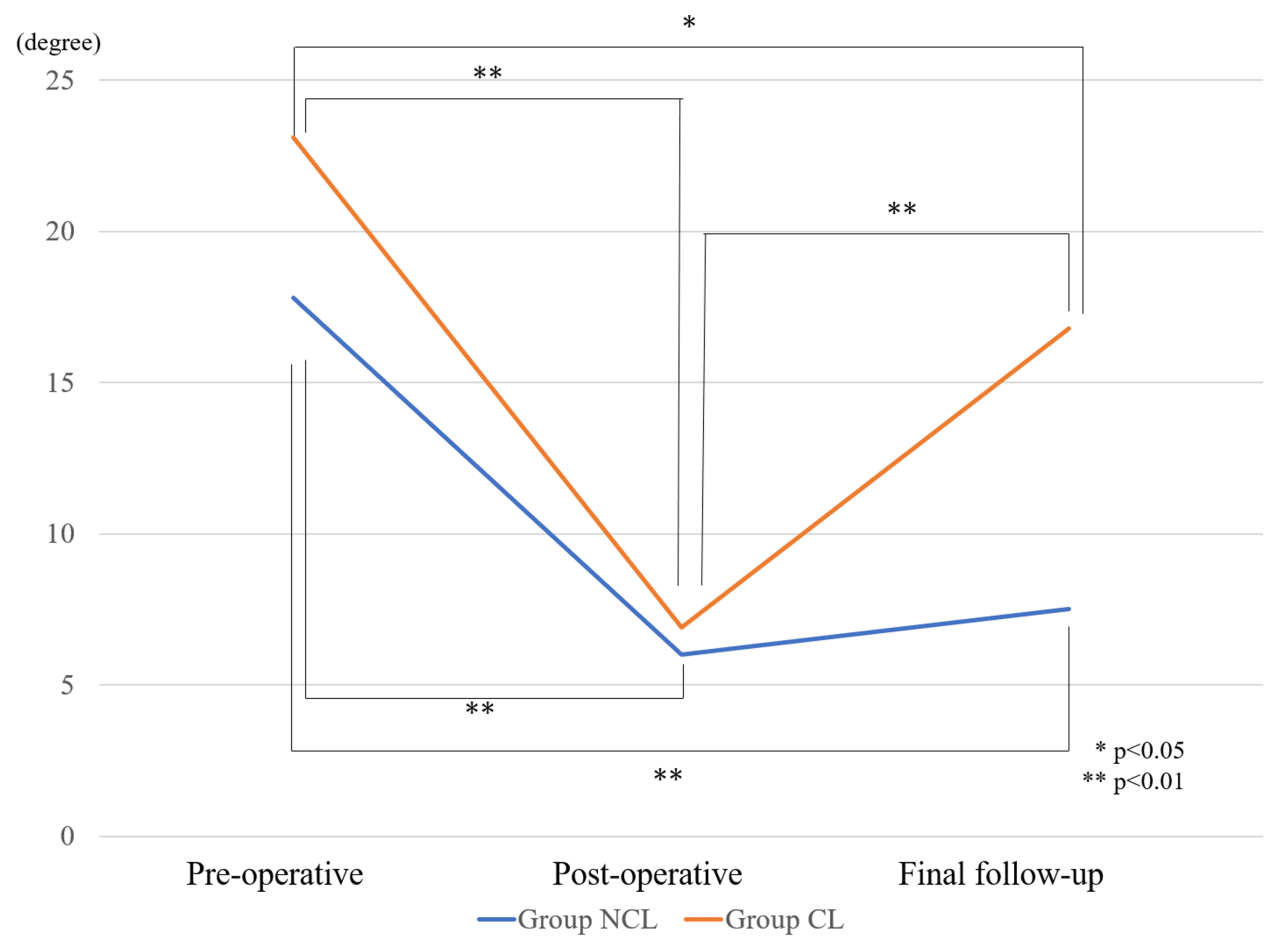
Vertebral wedge angle. Pre-operative, post-operative, and final follow-up. Statistical analysis was done using the Mann-Whitney U test. NCL: no correction loss, CL: correction loss.

**Figure 5:**
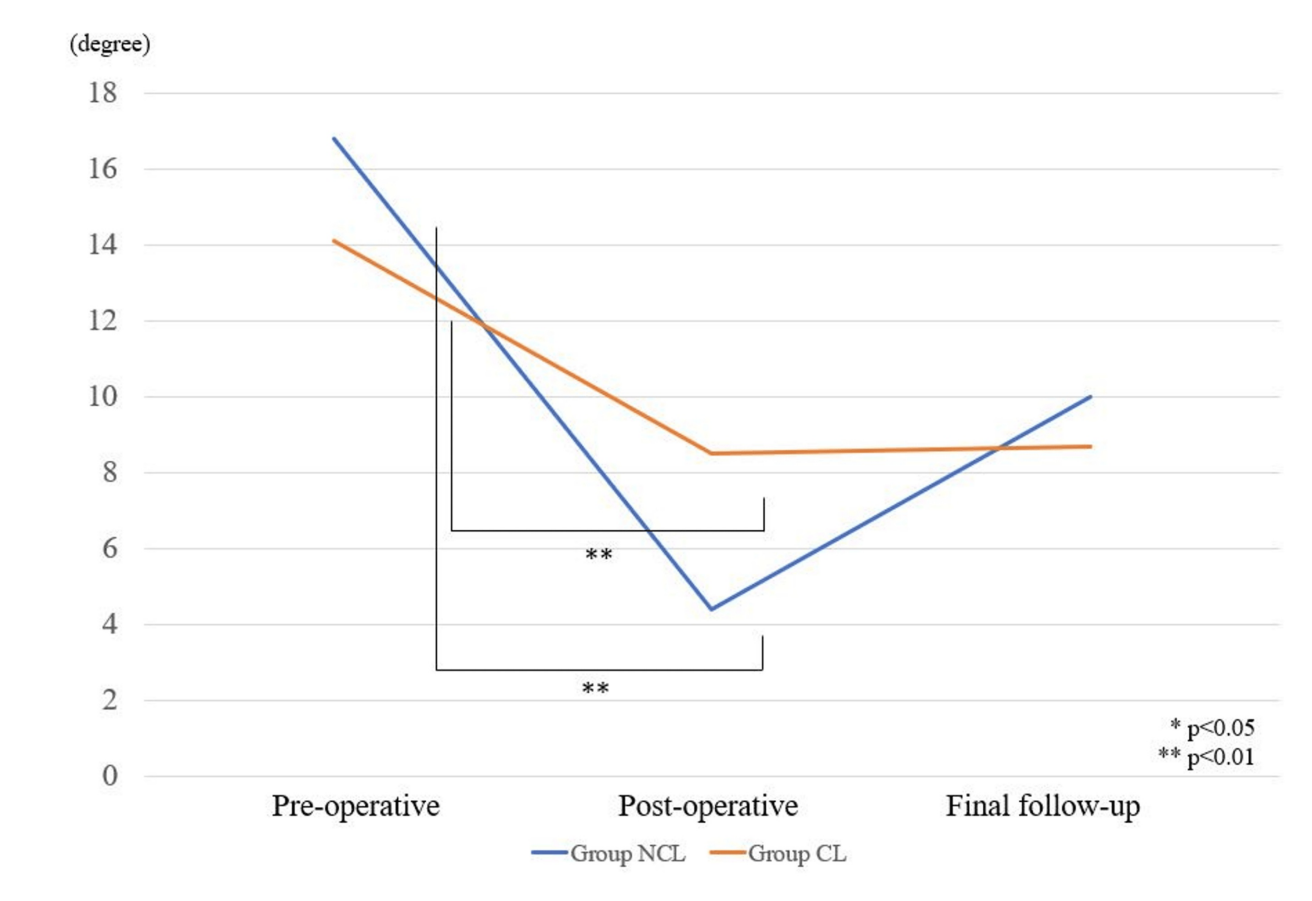
Focal kyphotic angle. Pre-operative, post-operative, and final follow-up. Statistical analysis was done using the Mann-Whitney U test.

All patients in our study achieved satisfactory correction in vertebral wedge angle and focal kyphotic angle post-operatively. The post-operative and final vertebral wedge angles were statistically improved in Group NCL (p<0.001) and maintained (p<0.001). The post-operative vertebral wedge angle was significantly improved in Group CL (p<0.001) but decreased at the final follow-up (p<0.001) (Figure 4). The post-operative and final focal kyphotic angles were statistically improved in Group NCL (p<0.001, p<0.001), and maintained the angle (Figure 5).

The pre-operative vertebral wedge angle of Group CL was significantly larger than that of Group NCL (23.1 ± 6.8, 17.8 ± 6.7 degrees, *p*=0.045). However, both groups' post-operative vertebral wedge angles were not statistically different (6.9 ± 3.5, 6.0 ± 3.2 degrees, *p*=0.539). The pre-operative focal kyphotic angle of Group CL was larger than that of Group NCL, but there was no statistically significant difference (24.8 ± 14.1, 16.8 ± 10.8, *p*=0.183) (Table 2).

**Table 2:** Radiological parameters. Mann-Whitney U test was used for all. NCL: no correction loss, CL: correction loss.

	Group NCL	Group CL	Statistical test	Statistical value	p value
Vertebral wedge angle (pre-operative)	17.8 ± 6.7	23.1 ± 6.8	Mann-Whitney U	39	0.045
Vertebral wedge angle (post-operative)	6.0 ± 3.2	6.9 ± 3.5	Mann-Whitney U	64	0.539
Vertebral wedge angle (final follow-up)	7.5 ± 3.0	16.8 ± 6.2	Mann-Whitney U	5	<0.0001
Correction gain	11.8 ± 7.3	16.2 ± 5.8	Mann-Whitney U	46.5	0.113
Correction loss	1.47 ± 2.3	9.9 ± 3.4	Mann-Whitney U	0	<0.0001
Focal kyphotic angle (pre-operative)	16.8 ± 10.8	24.8 ± 14.1	Mann-Whitney U	51	0.183
Focal kyphotic angle (post-operative)	4.4 ± 7.5	6.9 ± 8.5	Mann-Whitney U	60	0.404
Focal kyphotic angle (final follow-up)	10.0 ± 9.0	19.1 ± 8.7	Mann-Whitney U	34.5	0.024
Correction gain	12.4 ± 11.0	17.9 ± 9.8	Mann-Whitney U	55	0.267
Correction loss	5.6 ± 2.6	12.2 ± 6.9	Mann-Whitney U	23	0.0038

On MRI findings, four spinal cord injuries were observed in Group NCL and three in Group CL. Upper disc injury was more common than lower disc injury. Between the two groups, spinal cord, disc, and end plate injury status were not statistically different.

On pre-operative CT assessment, McCormack and Gaines load-sharing score, AO classification, pre-operative canal occupying ratio, and osteoporosis (HU) in both groups were not significantly different (Table 3).

**Table 3:** MRI and CT evaluation. Fisher exact test was used for MRI disc injury status, and AO classification. Mann-Whitney U test was used for McCormack, re-operative canal occupying ratio, and osteoporosis. NCL: no correction loss, CL: correction loss.

	Group NCL	Group CL	Statistical test	Statistical value	p value
MRI spinal cord injury status	Injured: 4, non-injured: 11	Injured: 3, non-injured: 7	Fisher exact	-	1
MRI Upper disc injury status	Grade 3: 13	Grade 3: 8	Fisher exact	-	1
MRI Lower disc injury status	Grade 3: 7	Grade 3: 4	Fisher exact	-	1
McCormack and Gaines load-sharing score	6.5 ± 1.7	6.7 ± 1.3	Mann-Whitney U	75	1
AO classification	A3: 11, A4: 4	A3: 9, A4: 1	Fisher exact	-	0.615
Pre-operative canal occupying ratio (%)	43.4 ± 12.0	43.1 ± 19.7	Mann-Whitney U	55	0.111
Osteoporosis: Hounsfield units (Axial)	155.6 ± 47.1	154.5 ± 62.5	Mann-Whitney U	71	0.222

Comparison of the surgical results between the two groups

The Group NCL had an average surgical time of 125.2 ± 46.3 minutes, and 97.3 ± 21.0 minutes were observed in the Group CL (*p*=0.149). Regarding blood loss, the Group NCL had an average blood loss of 145.7 ± 167.2 ml compared to 65.0 ± 88.0 ml in the Group CL. A comparison of surgical parameters between the two groups showed no significant differences in surgical time and average blood loss (*p*=0.149, *p*=0.083). The long/short construct was not significantly different between the two groups (*p*=0.467) (Table 4). Single rod breakage was observed in the CL group (*p*=0.4).

**Table 4:** Surgical parameters. Fisher exact test was used for surgical construct, implant failure. Mann-Whitney U test was used for surgical time, intraoperative blood loss. NCL: no correction loss, CL: correction loss.

	Group NCL	Group CL	Statistical test	Statistical value	p value
Surgical time (mins)	125.2 ± 46.3	97.3 ± 21.0	Mann-Whitney U	49	0.149
Intraoperative blood loss (ml)	145.7 ± 167.2	65.0 ± 88.0	Mann-Whitney U	44	0.083
Surgical construct	Short 10/long 5	Short 8/long 2	Fisher exact	-	0.659
Implant failure	0	1 (screw breakage)	Fisher exact	-	0.4

## Discussion

In our study, the percutaneous reduction and fixation of thoracolumbar burst fractures yielded good correction and maintenance of spinal alignment in all patients. Our criteria regarded correction loss of < 5 degrees in the final follow-up (one year) as a good radiological outcome. We analyzed various factors, and a large pre-operative vertebral wedge angle was the only significant factor for correction loss after percutaneous reduction and fixation. The NCL group had a 17.8 ± 6.7 degrees vertebral wedge angle of the fractured vertebra, as the CL group had a 23.1 ± 6.8 angle in our series. A large pre-operative vertebral wedge angle means greater initial deformity/severe vertebral collapse along with severe soft tissue and ligamentous injury [16]. Jang et al. analyzed 208 patients in their series and reported a large prevertebral wedge angle as the predictor of loss of correction gain after surgery [8]. Similarly, Costa et al. mentioned that a large kyphotic angle significantly affected surgical outcomes in their systematic analysis [17]. A combination of these factors, like a large vertebral wedge angle and soft tissue injury, makes it harder to restore and maintain normal alignment post-surgery.

The CL group had a correction gain of vertebral wedge angle greater than that of the NCL group, but not statistically different (16.2 ± 5.8, 11.8 ± 7.3 degrees, p=0.113). A large correction gain of defects and their maintenance increases mechanical stress on the fixation construct, eventually leading to correction loss in the follow-up [18]. Even the posterior tension band may be compromised at the fractured level when the fractured vertebra has a large vertebral wedge angle, leading to progressive loss of correction gained during the surgery [19]. After stabilization, the vertebrae undergo continuous axial loading, which can cause gradual subsidence and loss of correction [20]. Some studies suggested that excessive kyphosis correction, particularly if it involves a significant reduction of the fractured vertebral body, may lead to increased correction loss and instability in the long term [21]. This could be attributed to the disruption of the adjacent intervertebral disc and the potential for progressive disc degeneration [10].

Our findings showed no significant difference in the impact of osteoporosis between the CL and NCL groups. However, osteoporosis was reported as an important factor for correction loss [6]. Similarly, osteoporotic patients were prone to early instrumentation failure and progressive kyphosis, especially when treated with short-segment posterior fixation alone [19]. Pawar et al. demonstrated good clinical outcomes for osteoporotic burst fractures with MIS short-segment stabilization [22]. Modern MIS techniques provide better stability with improved implant stability as evidenced by the use of MIS dual threaded screw, described by Ishii et al. [23]. Moreover, the bone healing mechanism is intact even in osteoporosis [24]. A combination of our percutaneous reduction and fixation technique with better implants and modern medication might have reduced the significance of osteoporosis in our study.

The vertebral comminution (McCormack and Gaines score) was reported to be another critical factor for the correction loss for thoracolumbar fixation [9]. McCormack and Gaines's load-sharing classification was given for thoracolumbar burst fracture treated by open surgery, only focusing on vertebral body compression and bone loss [9]. This score > 6 was reported to be a critical factor in the study of Filgueira et al., but not significant in our study, as both groups had similar values (*p*=1) [25]. Several reports concluded that this load-sharing classification was unrelated to the correction loss. Park et al. reported that a severe score in the AO classification tends to have a poor prognosis in the long term [26]. The AO classification in our series didn’t impact the study's final outcome. Disc and endplate integrity potentially contribute to correction loss in long-term follow-up [12]. In our series, both groups had similar disc and endplate injury grades. Evidence suggests that patients with pre-operative endplate injuries do not necessarily exhibit greater correction loss, particularly when the posterior column and endplates are well supported [27]. They are not always poor indicators of surgical success [17].

In our study, both groups had a similar distribution of the two construct (long/short) types (*p*=0.659). The type of construct, whether long or short, is insignificant for subsequent correction loss with MIS surgery [13], intermediate screw, or intravertebral cement injection [28]. However, Hong et al. concluded that long-segment fixation offers enhanced stabilization, thereby improving alignment and spinal stability compared to short-segment fixation [29]. Biomechanically, in short, construct intermediate screws serve as the strategic push point, directing an anterior force that helps induce lordosis and minimizes the cantilever forces, reducing deformity to progress [30]. This suggests that both constructs offer comparable benefits in maintaining sagittal alignment over time. Therefore, if possible, we recommend the preferential use of short-segment fixation in future clinical practice.

Our study compared the two groups' surgical time and intraoperative blood loss, but there was no significant difference. This finding may be due to using the MIS procedure in both groups. However, in open-surgery patients, short-segment fixation was superior to long-segment fixation in terms of shorter surgical time and reduced intraoperative blood loss [11]. BMI and pre-operative neurological status were not significant in our study.

There are several limitations of this study. First, the sample size of both groups was small, and the follow-up period was relatively short. Second, there was a wide distribution of ages in the group`s potential for the confounding effect. Third, a dual-energy X-ray absorptiometry (DEXA) scan was not done to evaluate bone mineral density because of the emergent nature of the pathology.

## Conclusions

Percutaneous reduction and fixation for thoracolumbar burst fractures could result in acceptable correction and maintenance of spinal alignment. Reduction maneuvers in the percutaneous system are equally effective regardless of the type of construct. Patients with wedge angles of more than five degrees may benefit from adjuvant techniques.

An adequate understanding of the fracture morphology and patient-specific factors is essential before tailored surgical planning. The pre-operative large vertebral wedge angle was the only risk factor for correction loss. Short fixation, osteoporosis, and vertebral comminution were not significant risk factors in our study.
